# The Landmark Series: Surgical Management of Functioning and Non-Functioning Pancreatic Neuroendocrine Tumors

**DOI:** 10.1245/s10434-025-17390-x

**Published:** 2025-05-03

**Authors:** Joseph Tobias, Callisia N. Clarke, Alexandra Gangi, Xavier M. Keutgen

**Affiliations:** 1 Division of Surgical Oncology, Section of Endocrine Surgery, University of Chicago, Chicago, IL USA; 2 Division of Surgical Oncology, Medical College of Wisconsin, Milwaukee, WI USA; 3 Division of Surgical Oncology, Cedars Sinai Medical Center, Los Angeles, CA USA

**Keywords:** Pancreatic neuroendocrine tumor, Pancreatic neuroendocrine neoplasm, Active surveillance, Minimally invasive surgery, Liver debulking

## Abstract

Pancreatic neuroendocrine tumors (PNETs) are comparatively rare pancreatic malignancies that exhibit diverse biologic behavior, ranging from indolent tumors to widely metastatic cancers, with up to 15 % secreting hormones that cause symptoms. As a consequence, the management of PNETs is highly individualized and can include active surveillance of small (1–2 cm) and very small (< 1 cm) nonfunctioning tumors without worrisome features, parenchymal-sparing resection of appropriately located tumors, anatomic pancreatectomy and, in select cases, debulking of metastatic disease, particularly in the liver. This review synthesizes society recommendations and contemporary evidence guiding the surgical management of PNETs. Innovations in molecular profiling and systemic therapies hold promise to refine surgical algorithms for this heterogeneous tumor.

Pancreatic neuroendocrine tumors (PNETs or PanNETs), also referred to as pancreatic neuroendocrine neoplasms (PNENs), originate from islet cells of the endocrine pancreas and comprise 1–2 % of all pancreatic malignancies and 6 % to 10 % of all neuroendocrine neoplasms.^1–3^ The incidence of PNETs is gradually rising, in part due to high-resolution cross-sectional imaging with computed tomography (CT) and somatostatin-receptor-based positron emission tomography (PET DOTATATE), and is estimated to be 1 in 100,000 persons per year according to most recent population studies.^4^Although the majority of PNETs are non-functioning, up to 15 % secrete peptides that cause hormonal syndromes, including but not limited to insulin, gastrin, somatostatin, glucagon, vasoactive intestinal peptide, serotonin and, in rare cases, calcitonin, adrenocorticotropic hormone (ACTH) or parathyroid hormone-related protein (PTHrP).^5^

In contrast to poorly differentiated pancreatic neuroendocrine carcinomas (PNECs), PNETs are well-differentiated tumors that retain their neuroendocrine morphology.^6,7^ They are graded by Ki67 proliferation and mitotic indices using the WHO classification system (Table 1) and exhibit a range of biologic behavior, from indolent small tumors to widely metastatic cancers. Non-functioning PNETs (NF-PNETs) tend to be diagnosed at later stages of disease than their functioning counterparts and may have worse disease-specific survival.^8^ Approximately 5 % of PNETs occur in the setting of genetic syndromes such as multiple endocrine neoplasia type 1 (MEN1), Von-Hippel Lindau syndrome (VHL), neurofibromatosis type 1 (NF1), and tuberous sclerosis complex (TSC) (Table 2).

**Table 1:** The World Health Organization epithelial neuroendocrine neoplasm classification^7^

Morphology	Classification	Diagnostic criteria
Well-differentiated neuroendocrine tumors ( NETs)	Grade 1	< 2 mitoses/2 mm^2^ and/or Ki67 < 3 %
	Grade 2	2–20 mitoses/2 mm^2^ and/or Ki67 3–20 %
	Grade 3	>20 mitoses/2 mm^2^ and/or Ki67 > 20 %
Poorly differentiated neuroendocrine carcinomas (NECs)	Small cell	>20 mitoses/2 mm^2^ and/or Ki67 > 20 % with small or large cell cytomorphology and necrosis
	Large cell

**Table 2:** Management of PNETs in genetic syndromes^9,13–15^

Syndrome	Frequency (%)	Tumor characteristics	Management
MEN1	30–80	Located throughout the pancreas; most commonly NF-PNETs, followed by insulinomas and gastrinomas	Resection if functioning or > 2 cm, favoring pancreas-preserving approaches as most patients have multifocal disease and may require sequential resections
VHL	15–20	Always NF-PNETs	Resection if >3 cm, doubling time < 500 days or mutations in exon 3
NF1	0–10	Functioning and non-functioning tumors	Resection if functioning or > 2 cm
TSC	Rare	Functioning and non-functioning tumors	Resection if functioning or > 2 cm

PNET, pancreatic neuroendocrine tumor; MEN1, endocrine neoplasia type 1; NF, non-functioning; VHL, Von-Hippel Lindau syndrome; NF1, neurofibromatosis type 1; TSC, tuberous sclerosis complex

## Surgical Management

The surgical management of PNETs depends upon symptoms, tumor functionality, size, location within the gland, morphology, grade, extent of disease, and whether the tumor arose sporadically or in the setting of a genetic syndrome. Surgical resection of PNETs can vary from enucleation to anatomic pancreatectomy to debulking of metastatic disease. Poorly differentiated neuroendocrine carcinomas are infrequently considered for surgical resection given their aggressive behavior. Society guidelines (NANETS, ENETS, NCCN) recommend curative-intent surgery for all non-metastatic functioning PNETs (Table 3) and for non-functioning PNETs larger than 2 cm.^9–12^ The management of very small (<1 cm) and relatively small (1–2 cm) non-functioning PNETs as well as surgery for metastatic disease remain the subjects of ongoing debate.

**Table 3:** Management of functioning PNETs^9,16^

Tumor	Syndrome	Frequency (%)	Malignant (%)	MEN1 (%)	Management
Insulinoma	Hypoglycemia	40–60	< 10	4–5	Enucleation
Gastrinoma	Peptic ulcer disease	20–50	20–25	20–25	Pancreatectomy vs observation if MEN1 < 2 cm *and* medically controlled, or duodenotomy and enucleation if in the duodenum
VIPoma	Watery diarrhea, hypokalemia, achlorhydria	Rare	40–70	6	Pancreatectomy
Glucagonoma	Diabetes, necrolytic migratory erythema	Rare	50–80	1–20	Pancreatectomy
Somatostatinoma	Diabetes, cholelithiasis, diarrhea	Rare	> 70	10	Pancreatectomy, cholecystectomy

PNET, pancreatic neuroendocrine tumor; MEN1, endocrine neoplasia type 1

In this review, we note the nuances of the surgical management of well-differentiated PNETs and the latest evidence contributing to these debates. Because localized functioning PNETs are almost always managed with surgical resection, this review provides a summary of guideline recommendations for the extent of surgery in these cases (Table 3), but otherwise focuses on the complex surgical decision-making involved in treating non-functioning PNETs.

## Surgical Resection for NF-PNETs Larger than 2 cm

Surgery remains the curative-intent treatment for resectable NF-PNETs larger than 2 cm. Since the first Landmark Series, “Pancreatic Neuroendocrine Tumors” in *Annals of Surgical* Oncology in 2021, new retrospective studies have added to existing institutional and registry-based evidence in support of this recommendation.^17–20^ In 2022, Andreasi et al.^21^ published their systematic review and meta-analysis of 15 studies, including the study of 2754 patients with NF-PNETs submitted to curative-intent surgery. They reported a pooled overall disease recurrence of 21 %, with nodal involvement, tumor grade, and microvascular and perineural invasion as significant predictors of recurrence. These results were broadly replicated in two subsequent institutional studies. Møller et al.^22^ reported a 79 % 5-year disease-free survival rate in their 2023 study of 165 patients during a median follow-up period of 59 months. This was a diverse surgical cohort in which 75 % of the patients had localized disease, 19 % had regional disease, and 6 % had metastatic disease. Tumor size, Ki67 index, and nodal involvement significantly predicted recurrence. Comparably, in their 2024 single-center study of 223 surgical patients, Mukkala et al.^23^ reported a 76 % 5-year disease-free survival rate during a mean follow-up period of 47 months. The study included both functioning and non-functioning PNETs, as well as a subset of patients with metastatic disease. Predictors of recurrence included tumor size greater than 4 cm, Ki67 index above 20 %, nodal involvement, and perineural invasion.

By contrast, a multicenter study conducted by the Pancreatic Neuroendocrine Disease Alliance (PANDA) involving 1402 patients during a median follow-up period of 4.8 years found a higher 5-year disease-free survival rate, at 84.8 %, which may have been the result of more stringently excluding patients with metastatic disease.^24^ This contemporary evidence affirms that curative-intent surgical resection of PNETs is associated with long-term disease-free survival for a majority of patients, especially patients with low-grade, localized disease.

## Management of NF-PNETs Smaller than 2 cm

**Fig. 1:**
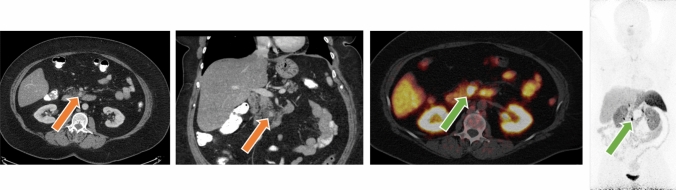
Computed tomography (CT) of the abdomen with intravenous (IV) contrast of a ~ 1 cm non-functioning PNET in the uncinate process (*orange arrows*) under active surveillance and without evidence of locoregional or distant metastases on DOTATATE PET CT (*green arrows*). PNET, pancreatic neuroendocrine tumor; PET, positron emission tomography

Current guidelines recommend pursuing active surveillance for sub-centimeter NF-PNETs and adopting an individualized approach to NF-PNETs 1 to 2 cm in size without worrisome features based on patient factors, such as surgical risk.^9,11^ Although multiple retrospective series have provided evidence that active surveillance is a safe alternative to surgical resection for NF-PNETs smaller than 2 cm for appropriately selected patients, prospective data have historically been lacking.^11,24–27^ Currently, results from two landmark prospective studies, the ASPEN and PANDORA trials, provide valuable evidence supporting the safety and feasibility of surveillance for well-selected cases (Fig. 1).

In 2022, Partelli et al.^25^ published an interim analysis of the ASPEN trial, a non-randomized controlled trial across 41 centers. The trial followed 500 patients with incidentally diagnosed, asymptomatic NF-PNETs ≤ 2 cm managed with either active surveillance or upfront surgical resection at the discretion of their treating surgical centers. Whereas 406 patients had active surveillance involving biannual CT or magnetic resonance imaging (MRI), 94 patients had upfront resection. Younger patient age, tumor size greater than 1 cm, and main pancreatic ductal dilation greater than 3 mm were associated with the decision to pursue upfront surgery. It is important to note that the study’s inclusion criteria permitted high-risk features and metastatic disease at presentation (e.g., 4 of 94 patients in the upfront surgery group had metastatic disease), making direct comparisons between study groups challenging.

During a median follow-up period of 25 months, 9 of 406 patients under surveillance had surgery due to measurable tumor growth, main pancreatic ductal dilation, or patient preference. Importantly, none of these patients had unresectable or metastatic disease as a consequence of surveillance, and the 397 patients who remained under surveillance did not experience significant disease progression. In the upfront surgery group, more aggressive pathologic features were present in one fifth of the patients, including a Ki67 index higher than 20 %, perineural invasion, microvascular invasion, nodal metastasis, and distant metastases. Although limited by its short follow-up time, the study’s findings represent higher-quality evidence that active surveillance may be safe and feasible for patients whose tumors lack high-risk features such as tumor growth and main pancreatic ductal dilation.

Adding to the rationale for active surveillance is a 2022 study by Ricci et al. estimating the risk of overtreatment of NF-PNETs ≤ 2 cm by comparing 285 surgical patients with 118 matched patients who were surveilled. The surgical patients had a slightly higher risk of death (0.9 per 1000 persons per year vs 0.2; *p *= 0.02), which was a significant albeit small excess hazard rate.

There also has been a recent interim analysis of the PANDORA trial, a prospective study enrolling patients across 10 centers into active surveillance of localized grades 1 or 2 NF-PNETs ≤ 2 cm without worrisome features.^26^ Worrisome features in this study were defined as biliary or pancreatic obstruction, pathologic lymph node enlargement, tumor infiltration into adjacent structures, and main pancreatic ductal dilation greater than 3 mm. In the 2021 analysis of 76 patients, 6 patients (8 %) had surgery during a median follow-up period of 17 months due to tumor growth of more than 0.5 cm per year or tumor size exceeding 2 cm, new radiographic lymphadenopathy, surgeon or patient preference, or diagnostic uncertainty. No patients under surveillance experienced radiographically detectable metastatic disease (sensitivity of CT, MRI, and PET CT is admittedly low for small metastases), and all patients with tumor growth who underwent surgery ultimately had resectable disease. However, one patient who experienced rapid tumor growth ultimately had peritoneal metastases diagnosed at surgery.

Taken as a whole, these prospective data support the safety and feasibility of active surveillance for localized NF-PNETs ≤ 2 cm for carefully selected patients because the majority of small tumors appear to remain indolent over time. High-risk features of small tumors continue to warrant surgical intervention given their association with potentially aggressive pathology, and prospective follow-up evaluation still is needed to characterize safety outcomes over the longer term.

## Molecular Profiling

Currently, tumor size, grade, Ki67 index, and other clinicopathologic variables are used as predictors of biologic behavior. However, molecular profiling is likely to become increasingly important for the prognostication of the biologic behavior of PNETS. Although routine biopsy of small and very small PNETs is not mandated by current guidelines, it is the view of the authors that molecular profiling likely will become part of clinicians’ processes to determine which tumors should undergo resection.

Notably, alterations in MEN1, ATRX/DAXX, ARX, PDX1, and the mTOR-signaling pathway have been investigated as individual biomarkers, and in a 2024 transcriptomic analysis by Greenberg et al.,^28^ which included tumors smaller than 2 cm in diameter, machine-learning was used to create an eight-gene panel predictive of the metastatic phenotype with relatively high sensitivity and specificity. Prospective independent validation of this panel is ongoing.^27–29^

## Parenchymal-Sparing Resection

Parenchymal-sparing resection, defined as tumor enucleation or, in rare cases, central pancreatectomy, has been shown to result in equivalent oncologic outcomes while reducing exocrine and endocrine insufficiency.^30–32^ Enucleation is appropriate for small PNETs without radiographic evidence of lymph node involvement at least 2 mm away from the main pancreatic duct and is particularly suited to small functioning tumors with low metastatic potential, such as insulinomas, or favorably located NF-PNETs that exceed the 2 cm threshold for active surveillance. In Bolm et al.’s ^33^ 2022 retrospective analysis of four institutional databases, 810 patients with sporadic NF-PNETs smaller than 3 cm were studied over a median follow-up period of 208 months. Of these patients, 221 who underwent parenchymal-sparing resection (either enucleation or central pancreatectomy) were compared with 221 matched patients who underwent anatomic resection. The study found that the two groups had comparable disease-specific and overall survival. Parenchymal-sparing resection was more frequently performed using a minimally invasive approach (32.6 % vs 13.6 %) and was associated with less blood loss and shorter operative times, although also with a trend toward higher rates of postoperative pancreatic fistula (POPF). In summary, although achieving negative margins is the goal of surgical resection, aggressive resection to obtain wider margins is not necessarily associated with improved outcomes.

## The Role of Lymphadenectomy

The role of lymphadenectomy in the surgery of PNETs remains controversial because no high-quality evidence exists to indicate that lymphadenectomy has an impact on survival. Current guidelines therefore differ in their recommendations for lymph node dissection. The North American Neuroendocrine Tumor Society (NANETS) recommends sampling 11 to 15 nodes during anatomic pancreatectomy for the purposes of tumor staging.^9^This extent of lymph node dissection is, in practice, achieved as a byproduct of resection rather than as a targeted effort. The NANETS guidelines do not advise performing an additional lymphadenectomy during parenchymal-sparing resection. These recommendations align with the European Neuroendocrine Tumor Society (ENETS) guidelines, which advise routine lymphadenectomy for tumors larger than 3 cm, the same size threshold that ENETS uses to recommend against enucleation.^11^ In contrast, the Delphi consensus of the Americas Hepato-Pancreato-Biliary Association (AHPBA) takes a more selective approach, recommending lymphadenectomy during anatomic resection only when preoperative imaging identifies suspicious regional nodes.^34^

This discrepancy between guidelines arises from the lack of clear evidence supporting the oncologic benefits of lymphadenectomy. In their analysis of National Cancer Database (NCDB) data, Mao et al.^35^ studied 2132 surgical PNET patients, 80 % of whom underwent lymphadenectomy with a median of nine nodes sampled, and found that lymphadenectomy was not associated with prolonged survival. In Conrad et al.’s^36^ Surveillance, Epidemiology, and End Results (SEER) database study of 982 patients, lymphadenectomy also did not correlate with altered survival at any tumor size.

Most recently, in a 2024 systematic review of 52 studies involving 24,608 patients, Clarke et al.^37^ found low-quality evidence for a correlation between lymph node metastasis and worse disease-specific and overall survival. However, only two studies directly evaluated the benefit of lymphadenectomy, and their findings were inconclusive. Given this equivocal evidence, the decision to pursue lymphadenectomy probably should be individualized, considering tumor size, location, and preoperative imaging.

## Minimally Invasive Surgical Approaches

Minimally invasive surgery (MIS) can be effectively used to treat PNETs at centers with expertise. Minimally invasive distal pancreatectomy for a range of pathologies has been shown in the most up-to-date systematic reviews and meta-analyses to enhance functional outcomes without compromising survival.^38^ A 2019 analysis from the US Neuroendocrine Tumor Study Group of 576 patients undergoing distal pancreatectomy demonstrated that laparoscopic and robotic approaches were associated with less blood loss, fewer serious complications, shorter hospital length of stay, and equivalent overall survival compared with open surgery.^39^

In a subsequent 2022 analysis of 1023 patients from the Pancreatic Neuroendocrine Disease Alliance (PANDA), minimally invasive surgery, including pancreaticoduodenectomy, distal pancreatectomy, and enucleation, was non-inferior to open surgery in terms of recurrence-free survival and overall survival.^40^ The highest quality of evidence corroborating these findings derives from the 2019 LEOPARD randomized controlled trial comparing MIS with open distal pancreatectomy, in which 65 % of resections involved PNETs. In the trial, a minimally invasive approach was associated with lower-volume blood loss and shorter recovery times.^41^ Regarding MIS pancreaticoduodenectomy, however, the 2019 LEOPARD-2 randomized controlled trial comparing laparoscopic with open pancreaticoduodenectomy was prematurely terminated because of a higher complication-related death rate in the laparoscopic group.^42^ To date, multicenter retrospective evidence indicates that the robotic Whipple procedure, when performed by high-volume robotic pancreas surgeons, results in equivalent mortality with a reduction in other major complications, but prospective data are lacking.^43^

## Surgery for Metastatic Disease

**Fig. 2:**
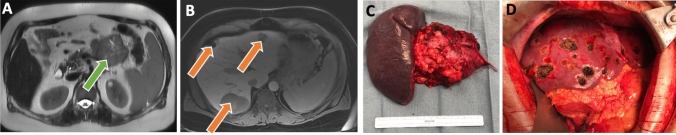
Magnetic resonance imaging (MRI) of the abdomen showing **A** a PNET in the tail of the pancreas (T2 sequence, *green arrow*) and **B** liver metastases (EOVIST phase, *orange arrows*). **C** surgical specimen and **D** liver after primary tumor resection and liver-debulking using parenchymal-sparing techniques and ultrasound-guided microwave ablation

One of the most debated aspects of the surgery of PNETs and of gastroenteropancreatic neuroendocrine tumors (GEP-NETs) more generally is the utility of primary tumor resection and liver-debulking or cytoreductive hepatectomy for metastatic disease (Fig. 2). Patients are candidates for debulking when they have no or limited extrahepatic and extra-abdominal disease, which can be detected with high sensitivity using preoperative DOTATATE PET CT, and when the treating surgeon can achieve a debulking threshold of 70–90 % of the neuroendocrine tumor metastasis burden.^9,44,45^

Large retrospective series during the past 30 years have consistently demonstrated that adequate liver-debulking using parenchymal-sparing techniques (wedge resection, enucleations, partial lobectomy, and thermal ablation) is associated with significant symptom improvement and prolonged survival.^45–50^ Conclusions that can be drawn from these studies are limited by a mixed evidence base that includes both small bowel neuroendocrine tumors (SBNETs) and PNETs, and by selection bias. However, prospective controlled data are practically and ethically challenging to obtain in this patient population.

In 2023, Gudmundsdottir et al.^45^ published one of the largest single-institution retrospective reviews of surgical outcomes after liver-debulking for GEP-NETs in 576 patients with 194 PNETs during 21 years. They found a PNET-specific median progression-free survival of 10 months and an overall survival of 113 months, which track with historical controls. Major complications occurred for 20 % of the patients, whereas the postoperative mortality rate was 1.6 %. Also published in 2023, Lee et al.’s^51^ retrospective series of liver-debulking outcomes among 53 patients at a single center demonstrated an even lower rate of major complications, at 13 %, and no cases of liver failure using parenchymal-sparing techniques and microwave ablations.

The rationale behind surgical debulking of metastatic disease is to decrease hormone levels in functional tumors and to “reset the clock” on hepatic parenchymal replacement by tumor, thereby delaying liver failure from overwhelming tumor burden.^52^ It is important to acknowledge, however, that liver-debulking remains controversial and is not always feasible. In these and other cases, interventional radiologic therapies with bland, chemo- or radio-embolization are used to treat the neuroendocrine tumor liver burden. Although the first randomized controlled trials investigating liver-directed therapy are underway, existing evidence remains low in quality, primarily supporting its role in radiographic response and symptom relief. A systematic review by Kanabar et al.,^53^ of 101 studies including 5545 patients with NETs, 30 % of which were PNETs, found that liver-directed therapy resulted in a pooled partial response rate of 36.6 %, a symptom relief rate of 55.2 %, and median progression-free survival of 18 months.

There has been developing interest in exploring whether debulking surgeries can improve the effectiveness of systemic therapies for patients with metastatic NETs. In particular, exploring the relationship between tumor volume, prognosis, and response to systemic therapy has led to recent investigations of how surgery might enhance radioligand therapy.^54,55^ In a 2021 retrospective study of 889 patients with metastatic GEP-NETs, Kaemmerer et al. found that patients who had their primary tumors resected and received at least one cycle of peptide receptor radionuclide therapy (PRRT) experienced longer progression-free and overall survival than patients who did not have surgery. Additionally, Tobias et al.^56^ in 2024 demonstrated that surgical debulking with primary tumor resection and liver-debulking, leading to a reduction in tumor volume, prolonged progression-free survival after treatment with ^177^Lutetium-DOTATATE in a cohort of 89 GEP-NET patients, 34 of whom had PNETs.^56^

Even more controversial is the value of primary tumor resection in the setting of unresectable metastatic disease. Studies using the SEER database have suggested both a disease-specific and overall survival advantage to primary pancreatic tumor resection, and a 2019 NCDB study of 14,510 patients with metastatic GEP-NETs, 6088 of whom had pancreatic primary tumors, also demonstrated prolonged survival.^57–60^ It is important to remember, however, that unlike small bowel NETs, pancreatic NETs are less likely to obstruct the gastrointestinal tract, bleed, or perforate, and that when PNETs are located within the head of the pancreas, a morbid procedure such as pancreaticoduodenectomy may be required for resection of the primary tumor. Additionally, biliary-enteric anastomosis increases the risk of infection after future liver-directed therapies.^61^ Therefore, most high-volume centers consider primary tumor resection for patients with PNETs and unresectable metastatic disease if the tumor is located in the body or tail of the pancreas, or if the primary tumor is causing symptoms that cannot be managed without resection, such as sinistral hypertension and upper gastrointestinal bleeding.^62^

## Perioperative Systemic Therapies

In parallel with the use of surgery to enhance systemic therapies, established and new systemic therapies for advanced PNETs are available that might impact surgical algorithms. Examples include the chemotherapies capecitabine and temozolomide (CAPTEM) in the 2022 E2211 randomized controlled trial, the multikinase inhibitors lenvatinib in the 2021 TALENT trial and cabozantinib in the 2024 CABINET trial, as well as ^177^Lutetium-DOTATATE PRRT in the 2024 NETTER-2 trial.^63–67^

To date, small-scale investigations have demonstrated that neoadjuvant radioligand therapy and/or chemotherapy may downstage disease that is borderline resectable due to vascular involvement or tumor encroachment on adjacent anatomy. In 2020, Squires et al.^68^ retrospectively reviewed 30 patients with locally advanced and metastatic PNETs who received neoadjuvant CAPTEM and found that 43 % of patients exhibited favorable radiographic responses that facilitated surgery. In 2021, Opalinksa et al.^69^ found that four of nine GEP-NET patients who had advanced disease managed with radioligand therapy as first-line treatment became operative candidates, and that R0 resection became possible for two of the patients. Additionally, one prospective controlled trial currently has investigated the safety of neoadjuvant radioligand therapy before surgery.^70^ The 2024 NEOLUPANET multicenter, single-arm phase 2 trial of patients with sporadic NF-PNETs harboring at least one high-risk feature (tumor size > 4 cm, Ki-67 index > 10 %, adjacent organ involvement or vascular invasion) enrolled 31 patients treated with four cycles of ^177^Lutetium-DOTATATE before surgery. In this trial, 60 % of the patients exhibited a partial response to PRRT, and 28 of the 31 patients ultimately underwent successful surgical resection with no resultant mortality, but with grade III complications occurring in 7 patients. Longer-term follow-up evaluation is ongoing to ascertain the effect of neoadjuvant radioligand therapy on recurrence rates after surgery.

## Conclusion

Taken together, the studies reviewed in this landmark series describe the evolving uses of surgery in the treatment of localized and metastatic pancreatic NET. On the one hand, they provide growing evidence for the safety and feasibility of avoiding surgery in a subset of carefully selected small NF-PNETs that may be comparatively indolent malignancies. On the other hand, they potentially expand the uses of and rationale for surgery combined with systemic therapies in the treatment of advanced and metastatic disease. Looking toward the future, molecular profiling likely will be incorporated into predicting recurrence as well as metastatic risk and response to therapy, and combining systemic therapies and surgery will transform the management of advanced disease. In the interim, the surgical management of PNETs remains complex and requires nuanced decision-making to balance the risks of surgery with the potential for harm from this unpredictable tumor.
